# RETRACTION: Resveratrol Derivative, Trans-3, 5, 4′-Trimethoxystilbene Sensitizes Osteosarcoma Cells to Apoptosis via ROS-Induced Caspases Activation

**DOI:** 10.1155/omcl/9847186

**Published:** 2025-02-27

**Authors:** Oxidative Medicine and Cellular Longevity

RETRACTION: Y. Feng, J. Clayton, W. Yake, J. Li, W. Wang, L. Winne, and M. Hong. “Resveratrol Derivative, Trans-3, 5, 4′-Trimethoxystilbene Sensitizes Osteosarcoma Cells to Apoptosis via ROS-Induced Caspases Activation,” *Oxidative Medicine and Cellular Longevity* vol. 2021, (2021): Article ID 8840692, 18 pages, https://doi.org/10.1155/2021/8840692.

The above article, published online on 26 March 2021 in Wiley Online Library (wileyonlinelibrary.com), has been retracted by agreement between John Wiley & Sons Ltd. and the journal Editor-in-Chief, Jeannette Vasquez-Vivar.

The retraction has been agreed following an investigation into concerns originally raised by Elizabeth Bik, Actinopolyspora biskrensis, and Tulipa Fosteriana on PubPeer (https://pubpeer.com/publications/4B54C4603D17F0D264C1C82E95BA24#5), which identified significant overlap with a previously published article from the same group of authors [1].

Figures 1–9 are entirely or partially identical to the Figures presented in [1], where they correspond to different cell types and treatments. In addition, the 143B and Saos-2 cells depicted in Figure 1b appear identical to images of MCF-7 cells from Figure 5c of [2]. Concerns have also been raised with the accuracy of 4 authors' listed affiliations.

The authors were informed of the decision to retract but remained unresponsive.
